# Update: Anti‐Amyloid Treatments for Alzheimer's Disease: Patients’ and Families’ Perception and Experience with Drug Consultation

**DOI:** 10.1002/alz70858_098187

**Published:** 2025-12-24

**Authors:** Allison Lapins, Kaitlyn Lucca, Joshua Gabriel Cahan, Sarah Welch, Darby J. Morhardt

**Affiliations:** ^1^ Northwestern University Feinberg School of Medicine, Chicago, IL, USA; ^2^ Beuhler Center for Health Policy and Economics, Northwestern University Feinberg School of Medicine, Chicago, IL, USA

## Abstract

**Background:**

New anti‐amyloid monoclonal antibody therapies (AATs) for Alzheimer's Disease (AD) are available but little is known about how treatment decisions are made by patients and their families. Drug consultations for these new therapies for AD are currently taking place at the Northwestern Medical Group's Neurobehavior and Memory Clinic with potentially eligible patients and their families. Study aims are: 1) measure patient and family perceptions, barriers and concerns of drug treatment following a formal consultation or informal discussion with a behavioral neurologist, and 2) gather data on the patient and family experience with the drug consultation as a method for making an informed decision.

**Methods:**

Participants potentially eligible for AATs and have a drug consultation with their behavioral neurologist will be asked to participate in our study survey. To be eligible for AATs, a patient must be in early stages of the disease and have biomarker evidence of AD. Interested participants complete the survey over the phone with a study team member. The survey asks questions aimed to measure interest in starting treatment before and after consultation, concerns and barriers surrounding the treatment, as well as overall experience with the drug consultation.

**Result:**

To date, 20 participants have completed the survey following consultation with a neurologist. 5 care partners and 15 persons living with Alzheimer's Disease (PLWAD) were interviewed. Preliminary results indicate 80% of survey participants reported being “somewhat” or “very” interested in starting treatment after drug consultation. The most frequently reported concerns were medical complications of therapy [larger brain bleeds, brain swelling, micro‐hemorrhages]. Those who reported a decrease in interest level had concerns that therapy would not be impactful on their daily life.

**Conclusion:**

Drug consultation with a behavioral neurologist on the new Alzheimer's Disease therapies serves as an important decision‐making aid for patients and families when considering AATs‐ identifying concerns and ensuring understanding of risks, benefits and overall impact of taking these newly available treatments. We plan to do additional data analysis to evaluate whether demographic factors, such as age, play a role in decision‐making.

